# (*Z*)-3-(1*H*-Indol-3-yl)-2-(3,4,5-tri­methoxy­phen­yl)acrylonitrile

**DOI:** 10.1107/S1600536812005855

**Published:** 2012-02-17

**Authors:** Narsimha Reddy Penthala, Sean Parkin, Peter A. Crooks

**Affiliations:** a Department of Pharmaceutical Sciences, College of Pharmacy, University of Arkansas for Medical Sciences, Little Rock, AR 72205, USA; b Department of Chemistry, University of Kentucky, Lexington, KY 40506, USA

## Abstract

In the title compound, C_20_H_18_N_2_O_3_, the C=C bond of the acrylonitrile group that links the indole and the 3,4,5-trimeth­oxy­phenyl rings has *Z* geometry, with dihedral angles between the plane of the acrylonitrile unit and the planes of the benzene and indole ring systems of 21.96 (5) and 38.94 (7)°, respectively. The acrylonitrile group is planar (r.m.s. deviation from planarity = 0.037 Å). Mol­ecules are linked into head-to-tail chains that propagate along the *b*-axis direction by bifurcated N—H⋯O inter­molecular hydrogen bonds, which form an *R*_1_^2^(5) motif between the indole NH group and the two meth­oxy O atoms furthest from the nitrile group.

## Related literature

For biological activity of similar acrylonitriles, see: Naruto *et al.* (1983 ▶); Ohsumi *et al.* (1998 ▶); Saczewski *et al.* (2004 ▶). For the mol­ecular structures of (*E*)-3-(benzo[*b*]thio­phen-2-yl)-2-(3,4,5-trimeth­oxy­phen­yl)acrylonitrile and (*Z*)-3-(benzo[*b*]thio­phen-2-yl)-2-(3,4-dimeth­oxy­phen­yl)acrylonitrile, see: Sonar *et al.* (2007 ▶). For the structure of (*Z*)-4-[3-(2,5-dioxo­imi­dazol­idin-4-ylidenemeth­yl)-1*H*-indol-1-ylmeth­yl]benzo­nitrile, see: Penthala *et al.* (2008 ▶).
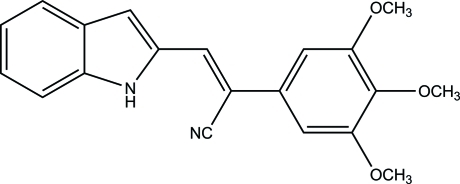


## Experimental

### 

#### Crystal data

C_20_H_18_N_2_O_3_
*M* *_r_* = 334.36
Monoclinic, 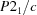

*a* = 11.3384 (4) Å
*b* = 21.1383 (8) Å
*c* = 6.9570 (3) Å
β = 93.610 (2)°
*V* = 1664.11 (11) Å^3^
*Z* = 4
Cu *K*α radiation
μ = 0.74 mm^−1^
*T* = 90 K
0.24 × 0.07 × 0.02 mm

#### Data collection

Bruker X8 Proteum diffractometer
Absorption correction: multi-scan (*SADABS*; Bruker, 2006 ▶) *T*_min_ = 0.843, *T*_max_ = 0.929
20812 measured reflections
2979 independent reflections
2679 reflections with *I* > 2σ(*I*)
*R*_int_ = 0.053

#### Refinement

*R*[*F* ^2^ > 2σ(*F* ^2^)] = 0.037
*wR*(*F* ^2^) = 0.097
*S* = 1.07
2979 reflections
233 parameters
H atoms treated by a mixture of independent and constrained refinement
Δρ_max_ = 0.26 e Å^−3^
Δρ_min_ = −0.20 e Å^−3^

### 

Data collection: *APEX2* (Bruker, 2006 ▶); cell refinement: *SAINT* (Bruker, 2006 ▶); data reduction: *SAINT*; program(s) used to solve structure: *SHELXS97* (Sheldrick, 2008 ▶); program(s) used to refine structure: *SHELXL97* (Sheldrick, 2008 ▶); molecular graphics: *XP* in *SHELXTL* (Sheldrick, 2008 ▶); software used to prepare material for publication: *SHELXL97* and local procedures.

## Supplementary Material

Crystal structure: contains datablock(s) global, I. DOI: 10.1107/S1600536812005855/nk2125sup1.cif

Structure factors: contains datablock(s) I. DOI: 10.1107/S1600536812005855/nk2125Isup2.hkl

Supplementary material file. DOI: 10.1107/S1600536812005855/nk2125Isup3.cml

Additional supplementary materials:  crystallographic information; 3D view; checkCIF report

## Figures and Tables

**Table 1:** Hydrogen-bond geometry (Å, °)

*D*—H⋯*A*	*D*—H	H⋯*A*	*D*⋯*A*	*D*—H⋯*A*
N1—H1N⋯O2^i^	0.851 (18)	2.075 (18)	2.8635 (15)	153.8 (16)
N1—H1N⋯O3^i^	0.851 (18)	2.301 (17)	2.9476 (15)	133.0 (15)

Symmetry code: (i) 
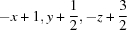
.

**Fig. 1.**
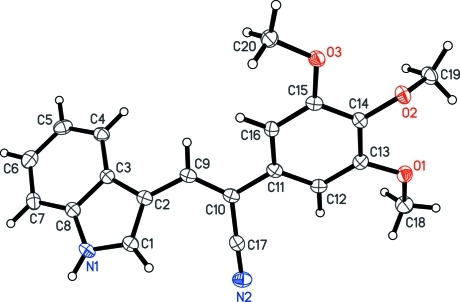
The asymmetric unit of (I). Displacement ellipsoids are drawn at the 50% probability level.

**Fig. 2.**
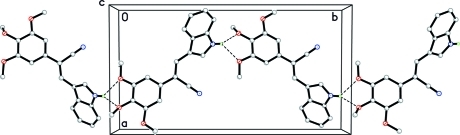
Bifurcated N–H···O bonding interactions (dashed lines) in the crystal structure of (I)

## supplementary crystallographic information

### Comment

A number of 2,3-diarylacrylonitrile analogs have been found to possess interesting biological properties such as spasmolytic (Naruto *et al.*, 1983), and cytotoxic activities (Ohsumi *et al.*, 1998 and Saczewski *et al.*, 2004). Previously, we have reported on the crystallographic data of benzothiophene acrylonitrile analogs (Sonar *et al.*, 2007). In continuation of the synthesis of structurally related analogs and to compare the structure–activity relationships of different substituted acrylonitrile analogs, we have now prepared the title compound, (I), by the reaction of indole-3-carbaldehyde with (3,4,5-trimethoxyphenyl)acetonitrile in methanolic sodium methoxide at reflux temperature. Recrystallization from methanol afforded yellow needles of (I) that were suitable for X-ray analysis. The X-ray studies revealed that the title compound is the *Z* isomer. The olefinic bond linking the indole ring and the 3,4,5-trimethoxyphenyl units has a planar atomic arrangement. The r.m.s. deviation from the mean plane passing through atoms of C1–C2–C3–C8–N1 is 0.0133 Å. The acrylonitrile group is planar (r.m.s. deviation from planarity is 0.037 Å). In the trimethoxyphenyl group, one methyl is essentially in the plane of the benzene ring and the three O atoms [deviation = 0.0253 (18) Å]. The middle methyl has the largest [deviation = 1.0600 (17) Å], while the methyl that is on the same side as the nitrile group is in between [0.3788 (19)Å out of plane]. Significant deviations from the ideal bond-angle geometry around the C*sp*^2^ atoms of the double bond are observed. The C1–C2–C3 and C1–C2–C9, C4–C3–C2 and C8–C3–C2 bond angles [105.88 (12)°, 127.93 (13)°, 134.18 (13)°, 106.90 (12)°, respectively] are distorted owing to steric hindrance around the double bond linking the two ring systems. Neither the indole ring nor the benzene ring of the 3,4,5-trimethoxyphenyl group is coplanar with the vinyl double bond, making dihedral angles of 38.94 (7)° and 21.96 (5)° respectively. Molecules are linked into head-to-tail chains that propagate along the *b* axis direction by bifurcated N—H···O intermolecular hydrogen bonds that form an *R*^2^_1_(5) motif between the indole NH and the two methoxy O atoms furthest from the nitrile group, as shown in Figure 2.

### Experimental

A mixture of indole-3-carbaldehyde (0.3 g, 2.06 mmol), and 2-(3,4,5-trimethoxyphenyl)acetonitrile (0.45 g, 2.17 mmol) were refluxed in methanolic 5% sodium methoxide solution for 5 hrs. The reaction mixture was cooled to room temperature and added to ice–cold water to afford a yellow crude solid, which was collected by filtration, washed with a 1:1 mixture of cold water and methanol, and suction–dried to afford the crude product. Crystallization during slow evaporation of methanol gave a yellow crystalline product of (*Z*)-3-(1*H*-indol-3-yl)-2- (3,4,5-trimethoxyphenyl)acrylonitrile that was suitable for X-ray crystallographic analysis. ^1^H NMR (CDCl_3_): δ 3.91 (s, 3H), 3.93 (s, 6H), 6.89 (s, 2H), 7.26–7.35 (m, 2H), 7.47–7.50 (d, 1H), 7.79 (s, 1H), 7.81 (s, 1H), 8.45–8.46 (d, 1H), 8.91 (bs, 1H, NH); ^13^C NMR (CDCl_3_): δ 56.57, 61.28, 102.92, 105.11, 106.03, 111.83, 112.91, 121.19, 121.75, 125.77, 127.47, 129.27, 130.94, 132.38, 138.19, 153.76.

### Refinement

H atoms were found in difference Fourier maps. H atoms attached to carbon were subsequently placed in idealized positions with constrained distances of 0.98 Å (RCH_3_) and 0.95 Å (C_sp2_H). The N—H hydrogen coordinates were freely refined, to a distance N—H = 0.851 (18) Å. *U*_iso_(H) values were set to either 1.2*U*_eq_ or 1.5*U*_eq_ (RCH_3_) of the attached atom.

### Crystal data

**Table:**

C_20_H_18_N_2_O_3_	*F*(000) = 704
*M**_r_* = 334.36	*D*_x_ = 1.335 Mg m^−^^3^
Monoclinic, *P*2_1_/*c*	Cu *K*α radiation, λ = 1.54178 Å
Hall symbol: -P 2ybc	Cell parameters from 9970 reflections
*a* = 11.3384 (4) Å	θ = 3.9–68.1°
*b* = 21.1383 (8) Å	µ = 0.74 mm^−^^1^
*c* = 6.9570 (3) Å	*T* = 90 K
β = 93.610 (2)°	Lath, yellow
*V* = 1664.11 (11) Å^3^	0.24 × 0.07 × 0.02 mm
*Z* = 4	

### Data collection

**Table:**

Bruker X8 Proteum diffractometer	2979 independent reflections
Radiation source: fine-focus rotating anode	2679 reflections with *I* > 2σ(*I*)
Graded multilayer optics monochromator	*R*_int_ = 0.053
Detector resolution: 5.6 pixels mm^-1^	θ_max_ = 68.1°, θ_min_ = 3.9°
φ and ω scans	*h* = −13→13
Absorption correction: multi-scan (*SADABS* in *APEX2*; Bruker, 2006)	*k* = −25→25
*T*_min_ = 0.843, *T*_max_ = 0.929	*l* = −7→8
20812 measured reflections	

### Refinement

**Table:**

Refinement on *F*^2^	Secondary atom site location: difference Fourier map
Least-squares matrix: full	Hydrogen site location: inferred from neighbouring sites
*R*[*F*^2^ > 2σ(*F*^2^)] = 0.037	H atoms treated by a mixture of independent and constrained refinement
*wR*(*F*^2^) = 0.097	*w* = 1/[σ^2^(*F*_o_^2^) + (0.0417*P*)^2^ + 0.7731*P*] where *P* = (*F*_o_^2^ + 2*F*_c_^2^)/3
*S* = 1.07	(Δ/σ)_max_ < 0.001
2979 reflections	Δρ_max_ = 0.26 e Å^−^^3^
233 parameters	Δρ_min_ = −0.20 e Å^−^^3^
0 restraints	Extinction correction: *SHELXL97* (Sheldrick, 2008), Fc^*^=kFc[1+0.001xFc^2^λ^3^/sin(2θ)]^-1/4^
Primary atom site location: structure-invariant direct methods	Extinction coefficient: 0.0016 (3)

### Special details

**Table:**

Geometry. All e.s.d.'s (except the e.s.d. in the dihedral angle between two l.s. planes) are estimated using the full covariance matrix. The cell e.s.d.'s are taken into account individually in the estimation of e.s.d.'s in distances, angles and torsion angles; correlations between e.s.d.'s in cell parameters are only used when they are defined by crystal symmetry. An approximate (isotropic) treatment of cell e.s.d.'s is used for estimating e.s.d.'s involving l.s. planes.
Refinement. Refinement of *F*^2^ against all reflections. The weighted *R*-value *wR* and goodness of fit *S* are based on *F*^2^. Conventional *R*-values *R* are based on *F*, with *F* set to zero for negative *F*^2^. The threshold expression of *F*^2^ > 2σ(*F*^2^) is used only for calculating *R*-factors(gt) *etc*. and is not relevant to the choice of reflections for refinement. *R*-values based on *F*^2^ are statistically about twice as large as those based on *F*, and *R*-values based on ALL data will be even larger.

### Fractional atomic coordinates and isotropic or equivalent isotropic displacement parameters (Å^2^)

**Table:**

	*x*	*y*	*z*	*U*_iso_*/*U*_eq_	
N1	0.29609 (11)	0.42543 (6)	0.78546 (16)	0.0203 (3)	
H1N	0.2929 (15)	0.4617 (8)	0.732 (2)	0.024*	
N2	0.68309 (12)	0.37056 (6)	0.89979 (17)	0.0251 (3)	
O1	0.91232 (9)	0.14011 (4)	1.03760 (14)	0.0220 (2)	
O2	0.79293 (9)	0.03501 (4)	0.91759 (13)	0.0202 (2)	
O3	0.56732 (8)	0.03817 (4)	0.81372 (14)	0.0203 (2)	
C1	0.38533 (13)	0.38300 (6)	0.77536 (19)	0.0195 (3)	
H1	0.4520	0.3883	0.7009	0.023*	
C2	0.36515 (12)	0.33101 (6)	0.88911 (18)	0.0177 (3)	
C3	0.25524 (12)	0.34334 (6)	0.97547 (18)	0.0172 (3)	
C4	0.18967 (13)	0.31113 (6)	1.1088 (2)	0.0206 (3)	
H4	0.2151	0.2712	1.1585	0.025*	
C5	0.08734 (14)	0.33867 (7)	1.1662 (2)	0.0253 (3)	
H5	0.0426	0.3174	1.2574	0.030*	
C6	0.04772 (14)	0.39732 (7)	1.0932 (2)	0.0266 (3)	
H6	−0.0241	0.4145	1.1335	0.032*	
C7	0.11116 (13)	0.43046 (7)	0.9640 (2)	0.0225 (3)	
H7	0.0850	0.4704	0.9153	0.027*	
C8	0.21522 (13)	0.40300 (6)	0.90769 (19)	0.0188 (3)	
C9	0.43611 (12)	0.27458 (6)	0.91389 (18)	0.0175 (3)	
H9	0.3947	0.2367	0.9389	0.021*	
C10	0.55447 (13)	0.26926 (6)	0.90571 (18)	0.0170 (3)	
C11	0.61787 (12)	0.20801 (6)	0.91199 (18)	0.0166 (3)	
C12	0.73704 (12)	0.20546 (6)	0.97301 (18)	0.0173 (3)	
H12	0.7781	0.2431	1.0103	0.021*	
C13	0.79597 (12)	0.14770 (6)	0.97940 (18)	0.0173 (3)	
C14	0.73641 (12)	0.09257 (6)	0.92416 (18)	0.0166 (3)	
C15	0.61699 (12)	0.09519 (6)	0.86363 (18)	0.0166 (3)	
C16	0.55781 (12)	0.15264 (6)	0.85555 (18)	0.0163 (3)	
H16	0.4768	0.1544	0.8118	0.020*	
C17	0.62542 (13)	0.32580 (6)	0.89881 (18)	0.0185 (3)	
C18	0.96763 (14)	0.19066 (7)	1.1428 (2)	0.0263 (3)	
H18A	0.9721	0.2277	1.0590	0.039*	
H18B	1.0476	0.1780	1.1892	0.039*	
H18C	0.9214	0.2012	1.2529	0.039*	
C19	0.84428 (13)	0.01136 (7)	1.0993 (2)	0.0242 (3)	
H19A	0.9256	0.0269	1.1196	0.036*	
H19B	0.8445	−0.0350	1.0971	0.036*	
H19C	0.7976	0.0262	1.2042	0.036*	
C20	0.44638 (13)	0.03775 (7)	0.7438 (2)	0.0227 (3)	
H20A	0.3972	0.0538	0.8440	0.034*	
H20B	0.4225	−0.0056	0.7100	0.034*	
H20C	0.4363	0.0648	0.6295	0.034*	

### Atomic displacement parameters (Å^2^)

**Table:**

	*U*^11^	*U*^22^	*U*^33^	*U*^12^	*U*^13^	*U*^23^
N1	0.0269 (7)	0.0156 (6)	0.0183 (6)	0.0011 (5)	−0.0004 (5)	0.0033 (5)
N2	0.0311 (7)	0.0229 (7)	0.0218 (6)	−0.0038 (5)	0.0048 (5)	−0.0011 (5)
O1	0.0174 (5)	0.0210 (5)	0.0270 (5)	−0.0010 (4)	−0.0031 (4)	−0.0036 (4)
O2	0.0221 (5)	0.0176 (5)	0.0202 (5)	0.0035 (4)	−0.0040 (4)	−0.0021 (4)
O3	0.0198 (5)	0.0160 (5)	0.0245 (5)	−0.0015 (4)	−0.0042 (4)	−0.0019 (4)
C1	0.0237 (8)	0.0196 (7)	0.0153 (6)	0.0002 (6)	0.0008 (6)	0.0000 (5)
C2	0.0222 (7)	0.0176 (7)	0.0131 (6)	−0.0018 (5)	−0.0017 (5)	−0.0013 (5)
C3	0.0196 (7)	0.0160 (7)	0.0153 (6)	−0.0018 (5)	−0.0030 (5)	−0.0031 (5)
C4	0.0240 (8)	0.0172 (7)	0.0205 (7)	−0.0027 (6)	−0.0007 (6)	−0.0008 (5)
C5	0.0271 (8)	0.0232 (7)	0.0262 (8)	−0.0059 (6)	0.0074 (6)	−0.0054 (6)
C6	0.0227 (8)	0.0252 (8)	0.0320 (8)	−0.0002 (6)	0.0034 (6)	−0.0095 (6)
C7	0.0230 (8)	0.0181 (7)	0.0257 (7)	0.0025 (6)	−0.0046 (6)	−0.0054 (5)
C8	0.0223 (7)	0.0169 (7)	0.0167 (6)	−0.0014 (5)	−0.0030 (6)	−0.0027 (5)
C9	0.0241 (8)	0.0166 (7)	0.0118 (6)	−0.0017 (5)	0.0006 (5)	−0.0002 (5)
C10	0.0238 (8)	0.0174 (7)	0.0098 (6)	−0.0011 (5)	0.0007 (5)	0.0003 (5)
C11	0.0225 (7)	0.0182 (7)	0.0095 (6)	0.0004 (5)	0.0035 (5)	0.0014 (5)
C12	0.0214 (7)	0.0173 (7)	0.0133 (6)	−0.0028 (5)	0.0014 (5)	−0.0006 (5)
C13	0.0184 (7)	0.0220 (7)	0.0116 (6)	−0.0010 (5)	0.0005 (5)	0.0007 (5)
C14	0.0206 (7)	0.0166 (7)	0.0125 (6)	0.0020 (5)	0.0005 (5)	−0.0003 (5)
C15	0.0218 (7)	0.0169 (7)	0.0110 (6)	−0.0023 (5)	0.0007 (5)	−0.0004 (5)
C16	0.0178 (7)	0.0195 (7)	0.0115 (6)	0.0011 (5)	−0.0001 (5)	0.0010 (5)
C17	0.0230 (7)	0.0202 (7)	0.0124 (6)	0.0035 (6)	0.0024 (5)	−0.0006 (5)
C18	0.0250 (8)	0.0240 (8)	0.0288 (8)	−0.0042 (6)	−0.0060 (6)	−0.0033 (6)
C19	0.0243 (8)	0.0216 (7)	0.0257 (7)	0.0022 (6)	−0.0062 (6)	0.0033 (6)
C20	0.0201 (8)	0.0210 (7)	0.0261 (7)	−0.0018 (6)	−0.0050 (6)	−0.0013 (6)

### Geometric parameters (Å, º)

**Table:**

N1—C1	1.3572 (19)	C7—H7	0.9500
N1—C8	1.3737 (19)	C9—C10	1.351 (2)
N1—H1N	0.851 (18)	C9—H9	0.9500
N2—C17	1.1499 (19)	C10—C17	1.4433 (19)
O1—C13	1.3649 (17)	C10—C11	1.4801 (18)
O1—C18	1.4188 (17)	C11—C12	1.392 (2)
O2—C14	1.3773 (16)	C11—C16	1.3980 (19)
O2—C19	1.4472 (17)	C12—C13	1.3910 (19)
O3—C15	1.3661 (16)	C12—H12	0.9500
O3—C20	1.4257 (17)	C13—C14	1.3890 (19)
C1—C2	1.3818 (19)	C14—C15	1.394 (2)
C1—H1	0.9500	C15—C16	1.3868 (19)
C2—C3	1.4409 (19)	C16—H16	0.9500
C2—C9	1.4430 (19)	C18—H18A	0.9800
C3—C4	1.4011 (19)	C18—H18B	0.9800
C3—C8	1.4113 (19)	C18—H18C	0.9800
C4—C5	1.380 (2)	C19—H19A	0.9800
C4—H4	0.9500	C19—H19B	0.9800
C5—C6	1.403 (2)	C19—H19C	0.9800
C5—H5	0.9500	C20—H20A	0.9800
C6—C7	1.377 (2)	C20—H20B	0.9800
C6—H6	0.9500	C20—H20C	0.9800
C7—C8	1.393 (2)		
			
C1—N1—C8	109.44 (12)	C12—C11—C10	120.23 (12)
C1—N1—H1N	125.7 (11)	C16—C11—C10	119.80 (13)
C8—N1—H1N	124.7 (11)	C13—C12—C11	119.93 (12)
C13—O1—C18	116.87 (11)	C13—C12—H12	120.0
C14—O2—C19	116.02 (10)	C11—C12—H12	120.0
C15—O3—C20	117.67 (11)	O1—C13—C14	115.30 (12)
N1—C1—C2	110.19 (12)	O1—C13—C12	124.47 (12)
N1—C1—H1	124.9	C14—C13—C12	120.23 (13)
C2—C1—H1	124.9	O2—C14—C13	122.10 (12)
C1—C2—C3	105.88 (12)	O2—C14—C15	118.06 (12)
C1—C2—C9	127.93 (13)	C13—C14—C15	119.76 (12)
C3—C2—C9	126.16 (12)	O3—C15—C16	124.87 (13)
C4—C3—C8	118.85 (13)	O3—C15—C14	114.77 (11)
C4—C3—C2	134.18 (13)	C16—C15—C14	120.36 (12)
C8—C3—C2	106.90 (12)	C15—C16—C11	119.73 (13)
C5—C4—C3	118.52 (13)	C15—C16—H16	120.1
C5—C4—H4	120.7	C11—C16—H16	120.1
C3—C4—H4	120.7	N2—C17—C10	177.68 (14)
C4—C5—C6	121.61 (13)	O1—C18—H18A	109.5
C4—C5—H5	119.2	O1—C18—H18B	109.5
C6—C5—H5	119.2	H18A—C18—H18B	109.5
C7—C6—C5	121.15 (14)	O1—C18—H18C	109.5
C7—C6—H6	119.4	H18A—C18—H18C	109.5
C5—C6—H6	119.4	H18B—C18—H18C	109.5
C6—C7—C8	117.25 (13)	O2—C19—H19A	109.5
C6—C7—H7	121.4	O2—C19—H19B	109.5
C8—C7—H7	121.4	H19A—C19—H19B	109.5
N1—C8—C7	129.82 (13)	O2—C19—H19C	109.5
N1—C8—C3	107.59 (12)	H19A—C19—H19C	109.5
C7—C8—C3	122.59 (13)	H19B—C19—H19C	109.5
C10—C9—C2	127.70 (13)	O3—C20—H20A	109.5
C10—C9—H9	116.1	O3—C20—H20B	109.5
C2—C9—H9	116.1	H20A—C20—H20B	109.5
C9—C10—C17	119.30 (12)	O3—C20—H20C	109.5
C9—C10—C11	123.60 (12)	H20A—C20—H20C	109.5
C17—C10—C11	117.04 (12)	H20B—C20—H20C	109.5
C12—C11—C16	119.97 (12)		
			
C8—N1—C1—C2	0.67 (16)	C9—C10—C11—C16	23.89 (19)
N1—C1—C2—C3	−0.36 (15)	C17—C10—C11—C16	−158.85 (12)
N1—C1—C2—C9	177.84 (13)	C16—C11—C12—C13	−0.58 (18)
C1—C2—C3—C4	−176.92 (15)	C10—C11—C12—C13	179.70 (11)
C9—C2—C3—C4	4.8 (2)	C18—O1—C13—C14	−163.42 (12)
C1—C2—C3—C8	−0.07 (15)	C18—O1—C13—C12	16.64 (18)
C9—C2—C3—C8	−178.31 (13)	C11—C12—C13—O1	−179.74 (11)
C8—C3—C4—C5	1.0 (2)	C11—C12—C13—C14	0.32 (19)
C2—C3—C4—C5	177.59 (14)	C19—O2—C14—C13	62.90 (16)
C3—C4—C5—C6	0.6 (2)	C19—O2—C14—C15	−120.39 (13)
C4—C5—C6—C7	−1.5 (2)	O1—C13—C14—O2	−3.80 (18)
C5—C6—C7—C8	0.7 (2)	C12—C13—C14—O2	176.14 (11)
C1—N1—C8—C7	179.00 (14)	O1—C13—C14—C15	179.55 (11)
C1—N1—C8—C3	−0.69 (15)	C12—C13—C14—C15	−0.51 (19)
C6—C7—C8—N1	−178.69 (14)	C20—O3—C15—C16	1.54 (18)
C6—C7—C8—C3	1.0 (2)	C20—O3—C15—C14	−177.79 (11)
C4—C3—C8—N1	177.88 (12)	O2—C14—C15—O3	3.54 (17)
C2—C3—C8—N1	0.46 (15)	C13—C14—C15—O3	−179.67 (11)
C4—C3—C8—C7	−1.8 (2)	O2—C14—C15—C16	−175.82 (11)
C2—C3—C8—C7	−179.26 (13)	C13—C14—C15—C16	0.97 (19)
C1—C2—C9—C10	31.1 (2)	O3—C15—C16—C11	179.48 (11)
C3—C2—C9—C10	−151.04 (14)	C14—C15—C16—C11	−1.22 (18)
C2—C9—C10—C17	9.7 (2)	C12—C11—C16—C15	1.03 (18)
C2—C9—C10—C11	−173.07 (12)	C10—C11—C16—C15	−179.25 (11)
C9—C10—C11—C12	−156.39 (13)	C9—C10—C17—N2	103 (4)
C17—C10—C11—C12	20.87 (17)	C11—C10—C17—N2	−74 (4)

### Hydrogen-bond geometry (Å, º)

**Table:**

*D*—H···*A*	*D*—H	H···*A*	*D*···*A*	*D*—H···*A*
N1—H1*N*···O2^i^	0.851 (18)	2.075 (18)	2.8635 (15)	153.8 (16)
N1—H1*N*···O3^i^	0.851 (18)	2.301 (17)	2.9476 (15)	133.0 (15)

Symmetry code: (i) −*x*+1, *y*+1/2, −*z*+3/2.
